# Paternal mtDNA and Maleness Are Co-Inherited but Not Causally Linked in Mytilid Mussels

**DOI:** 10.1371/journal.pone.0006976

**Published:** 2009-09-11

**Authors:** Ellen L. Kenchington, Lorraine Hamilton, Andrew Cogswell, Eleftherios Zouros

**Affiliations:** 1 Department of Fisheries and Oceans, Bedford Institute of Oceanography, Dartmouth, Nova Scotia, Canada; 2 Department of Biology, University of Crete, Heraklion, Crete, Greece; Northeastern University, United States of America

## Abstract

**Background:**

In marine mussels of the genus *Mytilus* there are two mitochondrial genomes. One is transmitted through the female parent, which is the normal transmission route in animals, and the other is transmitted through the male parent which is an unusual phenomenon. In males the germ cell line is dominated by the paternal mitochondrial genome and the somatic cell line by the maternal. Research to date has not allowed a clear answer to the question of whether inheritance of the paternal genome is causally related to maleness.

**Methodology/Principal Findings:**

Here we present results from hybrid crosses, from triploid mussels and from observations of sperm mitochondria in fertilized eggs which clearly show that maleness and presence of the paternal mitochondrial genome can be decoupled. These same results show that the female mussel has exclusive control of whether her progeny will inherit the mitochondrial genome of the male parent.

**Conclusions/Significance:**

These findings are important in our efforts to understand the mechanistic basis of this unusual mode of mitochondrial DNA inheritance that is common among bivalves.

## Introduction

The class Bivalvia is the only known group of organisms to contain species in which two mitochondrial genomes coexist in a stable state. The two mitochondrial genomes have different transmission routes. One mitochondrial genome, known as maternal or “female” (symbolized as F), is transmitted from mothers to both female and male progeny. The other mitochondrial genome, known as paternal or “male” (symbolized as M), is transmitted from males to their male offspring. The phenomenon has become known as doubly uniparental inheritance (DUI) of mitochondrial DNA (mtDNA) [1]. Currently, thirty-six species from seven different families of bivalves are known to have DUI [2]. The F mitochondrial genome cannot be passed from a male parent to its offspring, thus, its transmission does not differ from that of the typical animal mitochondrial genome. In contrast, the transmission of the M mitochondrial genome is strictly patrilinear. At present, there is no clearly established case of males, either wild-caught or produced from homospecific crosses, which do not contain M mtDNA [3]. Also, there is good evidence that sperm contains only M mtDNA [4]. DUI is, therefore, characterized by a strong linkage between M mtDNA inheritance and maleness [1], [5], [6], [7].

What might be the nature of this linkage? Could it be causative, meaning that in species with DUI the presence of the M mtDNA is needed for an embryo to develop into a male, a case analogous to the Y chromosome in mammals? Or might it be associative, meaning that development of maleness and M mtDNA inheritance are not causally linked, even though they are co-inherited? The question is undoubtedly one of crucial importance for our understanding of this exceptional mode of organelle inheritance. The most pertinent observations we have in our effort to answer this question are of two types. The first is the demonstration by Saavedra et al. [8] and Kenchington et al. [9] that in the blue mussel *Mytilus galloprovincialis* and its sibling species *M. edulis*, females can be grouped into three classes with regard to the sex ratio of their broods. There are females that produce almost exclusively daughters, females that produce sons in very high proportion and females that produce the two sexes in intermediate ratios. This property is independent of the male parent and is inherited as if determined by nuclear factors [9]. The second type of observation comes from the studies of Cao et al. [10] and Cogswell et al. [11], who have labeled sperm mitochondria of *Mytilus edulis* and observed their fate in eggs collected from mothers known to produce female-biased and male-biased offspring. They found that in the first type of eggs, the sperm mitochondria segregate at random among the blastomeres following division of the fertilized egg. In the second type of eggs, all or most of the sperm mitochondria form an aggregate that is located very close to the cleavage furrow, as if attached to the cell membrane. In successive divisions, the aggregate is found only in one blastomere. At the four-cell stage this blastomere is clearly distinguishable from the other three, and is known to give rise to the cell line which, among other tissues, will produce the germ cells [12]. The same behavior of sperm mitochondria was observed in *M. galloprovincialis* by Obata and Komaru [13]. These observations suggest a way through which all or most sperm mitochondria find their way into the male gonad. They also provide an explanation for the distribution of the M mtDNA in adult mussels. In females, the M mtDNA may not be detected or, more often, it may be detected as a small minority in various somatic tissues. In males, the M mtDNA dominates the gonad, but it may also be found as a small minority in somatic tissues that are dominated by the F mtDNA [14], [15].

It may appear unlikely that there could be a cause-effect relationship between entrance and domination of the gonad by the paternal mtDNA and subsequent development of the embryo into a male individual. Yet there are several reasons why this possibility cannot be dismissed. One reason is that mussels and bivalves in general, have no secondary sexual characteristics: a male mussel is a mussel with a male gonad and the same applies to females - and this is why mussels with undeveloped gonads cannot be sexed through external examination. A second reason is that we know nothing about sex determination in mussels, apart from the fact that there have been no sex chromosomes identified [16] and that in triploid mussels the development of the gonad resembles that of males [17]. The third reason is more subtle and relates to DUI. In *Mytilus edulis* and in *M. galloprovincialis*, there exists a third mitochondrial genome, in addition to the F and the M. This mitochondrial genome, designated as C, has a primary sequence very similar to the F mitochondrial genome, but it has a compound control region, consisting of the control region of the F mitochondrial genome in which there has been an insertion of three copies of the control region of the M mitochondrial genome [18]. What makes the C mitochondrial genome particularly interesting is that it is paternally transmitted. From the fact that it differs from the maternal mitochondrial genome only in that it contains the paternal control region, one may hypothesize that the insertion of the paternal control region has reversed the transmission route of the mitochondrial genome [19] (which is the reason these types of mitochondrial genomes have been called “masculinized” mitochondrial genomes). Burzinski et al. [20], [21] have also observed masculinized mitochondrial genomes in *Mytilus trossulus* from the Baltic Sea and proposed a similar hypothesis. The possibility that sequences of the mitochondrial genome are part of the mechanism that determines the mitochondrial genome's transmission route reinforces the hypothesis that in mussels sex determination and mitochondrial DNA inheritance might be causally linked.

It is important to ask what sort of evidence, if produced, would uncouple sex determination and paternal mtDNA inheritance in species with DUI. The occasional presence of the M mitochondrial genome in females cannot be such evidence, because it may be explained as a byproduct of the dispersed pattern of sperm mitochondria in eggs that have already been determined to develop into females. Several surveys of wild populations reported the presence of males that lacked the M mtDNA [22], [23], but this conclusion might be wrong for technical reasons. Males that apparently did not contain an M mitochondrial genome could have had a “masculinized” mitochondrial genome, which the assay used could not identify. When a proper assay was used in such studies, most males that lacked a typical M mitochondrial genome were found to carry a masculinized paternally inherited mitochondrial genome [24]. Also, the M mitochondrial genome accumulates mutations at a high rate [25] which may obliterate a primer recognition site. In this case the PCR assay will not give a product from the M mitochondrial genome of a male and the male would be classified as having only the F mitochondrial genome. This was the case in the study of *Mytilus galloprovincialis* crosses by Saavadra et al. [8], who reported that sons of a specific male did not contain a paternal mitochondrial genome. In a subsequent study, the authors used a second PCR assay and found that these sons did in fact contain the paternal mitochondrial genome of their father [3]. Specially designed crosses appear to be a safer way to address the question of a functional connection between maleness and presence of a paternally inherited mtDNA than surveys of natural populations. In such a study, Wood et al. [26] produced pure and hybrid crosses between the sibling species *M. edulis* and *M. galloprovincialis*. They scored individual larvae at the age of 3 hours and 72 hours post-fertilization for presence of their male parent's M mitochondrial genome. They observed significant differences in the proportion of larvae with this mitochondrial genome between pure and hybrid crosses sharing the same female parent. From this Wood et al. [26] concluded that hybridization disrupted DUI. The progeny's sex could not be checked at this age, so the nature of the disruption could not be identified. The hybridization may have affected the sex-ratio among the progeny of the female, but could have maintained the linkage between maleness and presence of the paternal mitochondrial genome. Alternatively, it could have affected the transmission of the M mitochondrial genome, but maintained the same sex-ratio.

Here we provide clear evidence that in the marine mussel, *Mytilus edulis*, sex determination and mitochondrial DNA inheritance are separate processes. We have combined sex scoring, mitochondrial DNA scoring and observations on the aggregation pattern of sperm mitochondria in fertilized eggs in progeny from pure and hybrid crosses. We also applied the same three assays in diploid and triploid progeny from the same females. Hybridization and triploidization were employed because previous studies suggested they may interfere with sex determination and mtDNA inheritance in mussels [7], [23], [17]. All these experiments were performed using females that produce either daughters or mostly sons. We have obtained a clear result: a female's control over presence or absence of the sperm's mtDNA in her offspring is maintained, whether the female is crossed to a male of her own or another species. Also, this control is not affected by the induction of triploidy in her progeny. In contrast, the sex ratio among progeny is seriously affected by hybridization and level of ploidy. These findings should be relevant in any effort to understand the molecular mechanism of DUI and sex determination in species with this unusual system of mtDNA inheritance.

## Results

### Nuclear and Mitochondrial Characterization of Parents

Twenty-two adult mussels were used as parents for the controlled crosses analyzed here: 12 females and 10 males (Table 1). All female and six of the male parents were confirmed to be *M. edulis* by the assay of two nuclear markers (Table 1). One male was identified as *M. trossulus* (01WM01) at both markers, and two males [01WM05 and (02WF01x01WM05)1] scored as *M. trossulus* at one nuclear locus and as *M. edulis* x *M. trossulus* hybrid at the other. A third male, (02WF01x01WM05)5, was identified as *M. trossulus* at both markers, however, the brother of this animal, (02WF01x01WM05)1, was a hybrid and so this animal was also classed as a hybrid. Male 01WM05 most likely resulted from of a backcross of an *M. edulis* x *M. trossulus* F_1_ hybrid to *M. trossulus* and, therefore, its nuclear genome is ¾ *M. trossulus* and ¼ *M. edulis*. The sperm of this individual carried the *M. trossulus* parental mtDNA type (see below), from which we conclude that its male parent was *M. trossulus*.

**Table 1:** Species and Mitotype Characterizaton of *Mytilus edulis* Parents used in Controlled Crosses.

Code of Individuals Used in Controlled Crosses	Sex of Individual	Species Identification: Nuclear Marker ITS	Species Identification: Nuclear Marker Glu-5′	Species Designation	Mitotype Identification: COIII/*Eco*RI	Mitotype Identification: COIII/*Acc*I	Mitotype Identification: Control Region/ssFdI	Mitotype Identification: Control Region/ssMdI	Use in Study
X102E	Female	M. edulis	M. edulis	M. edulis	Fed-1	NA	+	−	C,H,T
X102H	Female	M. edulis	M. edulis	M. edulis	Fed-1	Uncut	+	−	C,H
X102K	Female	M. edulis	M. edulis	M. edulis	Fed-1	Uncut	+	−	C,H,T
X102N	Female	M. edulis	M. edulis	M. edulis	Fed-1	Uncut	+	−	C,T
X102Q	Female	M. edulis	M. edulis	M. edulis	Fed-1	Uncut	+	−	C,T
X102R	Female	M. edulis	M. edulis	M. edulis	Fed-1	Uncut	+	−	C,H,T
X102S	Female	M. edulis	M. edulis	M. edulis	Fed-1	Uncut	+	−	C,H,T
(98Ax98WM8)A	Female	M. edulis	M. edulis	M. edulis	Fed-1	Uncut	+	−	C,H,T
(98Ax98WM8)B	Female	M. edulis	M. edulis	M. edulis	Fed-1	Uncut	+	−	C,H,T
(98Ax98WM8)C	Female	M. edulis	M. edulis	M. edulis	Fed-1	Uncut	+	−	H,T
98A	Female	M. edulis	M. edulis	M. edulis	Fed-1	Uncut	NA	−	C,H,T
(X102Cx98WM4)A	Female	M. edulis	M. edulis	M. edulis	Fed-1	Uncut	+	−	C,T
00WM2	Male	M. edulis	M. edulis	M. edulis	Uncut	Med-1	−	+	C,T
Z101C	Male	M. edulis	M. edulis	M. edulis	Uncut	Med-1	−	+	C,T
(98AxZ101A)1	Male	M. edulis	M. edulis	M. edulis	Uncut	Med-1	−	+	C,T
(98AxZ101C)2	Male	M. edulis	M. edulis	M. edulis	Uncut	Med-1	−	+	C,T
(98AxZ103)2	Male	M. edulis	M. edulis	M. edulis	Uncut	Med-1	−	+	C,T
J17F	Male	M. edulis	M. edulis	M. edulis	Uncut	Med-1	−	+	C,T
01WM01	Male	M. trossulus	M. trossulus	M. trossulus	Mtr-1	Uncut	−	−	H
(02WF01x01WM05)1	Male	M. trossulus	Hybrid	Hybrid	Mtr-1	Uncut	−	−	H,T
(02WF01x01WM05)5	Male	M. trossulus	M. trossulus	Hybrid	Mtr-1	Uncut	−	−	H,T
01WM05	Male	M. trossulus	Hybrid	Hybrid	Mtr-1	Uncut	−	−	H

NA: not assessed, C: control crosses (*M. edulis x M. edulis* for the production of pure-species diploids), H: hybrid crosses [(M. edulis x *M. trossulus* or *M. edulis* x (*M. edulis* x *M. trossulus* hybrid)], T: triploid crosses (M. edulis x M. edulis, from which a batch of eggs was used to produce triploids), Fed-1: the COIII mtDNA digestion pattern typical of the maternal *M. edulis* genome, Med-1: the COIII mtDNA digestion pattern typical of the paternal *M. edulis* genome, Mtr-1: the COIII mtDNA digestion product typical of the *M. trossulus* paternal genome.

We also scored the mtDNA COIII mitotype contained in the gametes of each parent (Table 1). In the eggs from all *M. edulis* females we detected only the F mitochondrial genome typical of *M. edulis* (symbolized as Fed-1) and in the sperm from all *M. edulis* males we detected only the M mitochondrial genome typical of the species (symbolized as Med-1), as expected. The *M. trossulus* male and the three hybrid males contained the typical *M. trossulus* M mtDNA (symbolized as Mtr-1). Therefore, none of the animals with hybrid nuclear genomes contained the M genome of *M. edulis*.

### Crosses

The parents listed in Table 1 were used to produce different types of crosses. These were *M. edulis* x *M. edulis* crosses (hereafter referred to as control crosses, C), *M. edulis* x *M.trossulus* or *M. edulis* x *M. hybrid* crosses for the production of hybrids (hereafter referred to as hybrid crosses, H) and *M. edulis* x *M. edulis* or *M. edulis x M. hybrid* crosses in which the eggs were treated with cytochalasin B for the production of triploids (hereafter referred to as triploid crosses, T). In total, we have produced 42 crosses. The crosses were categorized as “female-biased” (26 crosses, Table 2) and “male-biased” (16 crosses, Table 3) according to whether the mother produced female-biased or male-biased progeny. This designation was based on previous spawning history and the results of sexing the progeny of controlled crosses. The progeny from each cross were characterized as pure-diploid (PD) if they resulted from a *M. edulis* x *M. edulis* cross, as hybrid-diploid (HD) if they resulted from a *M. edulis* x *M.trossulus* or *M. edulis* x *M. hybrid* cross, as pure-triploid (PT) if they resulted from a *M. edulis* x *M. edulis* cross that was treated with catachalasin B and as hybrid-triploid if they resulted from a *M. edulis* x *M. hybrid* cross treated with cytochalasin B. An offspring was scored as “female”, “male” or “hermaphrodite” according to gonad development, as described in the Materials and Methods. It was also scored for its mtDNA content and characterized as “F” or “F+M” if it contained only the maternal mitochondrial genome or both the maternal and paternal genomes, respectively (Tables 2 and 3).

**Table 2:** Number of Progeny by Sex and Mitotype from Pure and Hybrid Crosses of Diploid and Triploid *Mytilus* Produced from Mothers Known to Produce Female-Biased Progeny.

Female Parent (Dam)	Male Parent (Sire)	Cross Type	Progeny Type	Sex Female/F mtDNA	Sex Female/F+M mtDNA	Sex Male/F mtDNA	Sex Male/F+M mtDNA	Sex Herm./F mtDNA	Sex Herm./F+M mtDNA
X102E	Z101C	ExE	PD	23	0	0	0	2	0
	Z101C	ExE	PT	0	0	22	0	0	0
	01WM01	ExT	HD	0	0	6	0	2	0
	(02WF01x01WM05)1	ExH	HD	0	0	13	0	2	0
	(02WF01x01WM05)1	ExH	HT	0	0	10	0	0	0
	01WM05	ExH	HD	11	0	14	0	5	0
X102H	Z101C	ExE	PD	30	0	0	0	0	0
	(02WF01x01WM05)1	ExH	HD	1	0	17	0	3	0
	01WM05	ExH	HD	19	0	9	0	2	0
X102K	Z101C	ExE	PD	29	0	0	0	1	0
	J17F	ExE	PD	10	0	0	0	0	0
	J17F	ExE	PT	0	0	16	0	0	0
	(02WF01x01WM05)5	ExH	HD	18	0	6	0	5	0
	(02WF01x01WM05)5	ExH	HT	0	0	6	0	0	0
	(02WF01x01WM05)1	ExH	HD	0	0	9	0	2	0
	01WM05	ExH	HD	33	0	18	0	17	0
X102Q	00WM2	ExE	PD	6	0	0	0	0	0
	00WM2	ExE	PT	0	0	24	0	0	0
X102R	00WM2	ExE	PD	30	0	0	0	0	0
	00WM2	ExE	PT	0	0	25	0	0	0
	(02WF01x01WM05)1	ExH	HD	17	0	4	0	9	0
	(02WF01x01WM05)1	ExH	HT	0	0	26	0	0	0
X102S	J17F	ExE	PD	10	0	0	0	0	0
	J17F	ExE	PT	0	0	20	0	0	0
	(02WF01x01WM05)5	ExH	HD	13	0	7	0	10	0
	(02WF01x01WM05)5	ExH	HT	0	0	15	0	1	0

Sex is scored as female, male or hermaphrodite. mtDNA content is scored as F (only maternal) or F+M (maternal and paternal). E: *M. edulis*, T: *M. trossulus*; P: pure-species, H: hybrid; D: diploid, T: triploid, Herm.: hermaphrodite.

**Table 3:** Number of Progeny by Sex and Mitotype from Pure and Hybrid Crosses of Diploid and Triploid *Mytilus* Produced from Mothers Known to Produce Male-Biased Progeny.

Female Parent (Dam)	Male Parent (Sire)	Cross Type	Progeny Type	Sex Female/F mtDNA	Sex Female/F+M mtDNA	Sex Male/F mtDNA	Sex Male/F+M mtDNA	Sex Herm./F mtDNA	Sex Herm./F+M mtDNA
X102N	(98AxZ103)2	ExE	PD	1	0	1	9	3	0
	(98AxZ103)2	ExE	PT	0	0	0	6	0	0
98A	Z101C	ExE	PD	3	0	0	27	0	0
	00WM2	ExE	PD	1	0	1	12	2	0
	00WM2	ExE	PT	0	0	1	7	0	0
	01WM01	ExT	HD	0	0	6	14	0	0
(98AxWM8)A	Z101C	ExE	PD	4	0	0	25	0	1
	Z101C	ExE	PT	0	0	4	6	0	0
	01WM05	ExH	HD	0	0	0	11	1	0
	(02WF01x01WM05)1	ExH	HT	0	0	7	10	0	0
(98AxWM8)B	Z101C	ExE	PD	0	0	0	4	0	0
	Z101C	ExE	PT	0	0	0	6	1	1
	01WM05	ExH	HD	0	0	0	2	0	0
(98AxWM8)C	(02WF01x01WM05)1	ExH	HD	0	0	0	8	0	0
	(02WF01x01WM05)1	ExH	HT	0	0	2	6	0	0
	01WM05	ExH	HD	0	0	0	3	0	0

Notation as for Table 2.

### Sex and Mitotype of Progeny from Hybrid Crosses with Female-biased Mothers

Ten crosses belonged to this category (crosses HD, Table 2). The five females used for the production of these crosses were also crossed to *M. edulis* (crosses PD, 7 crosses, Table 2). In crosses with the same dam the sex ratio was very different between those sired by an *M. edulis* male (cross type PD) and those sired by an *M. trossulus* or *M. hybrid* male (cross type HD). In the first type of crosses no male progeny were found. In the second type males were common, averaging 38%. The sex distribution was also different among hybrid crosses sharing the same mother or father. The chi-square test for homogeneity was significant in all these tests except for one [three crosses dammed by X102E, P = 0.02, two crosses dammed by X102H, P = 0.001, three crosses dammed by X102K, P = 0.001; three crosses sired by 01MW05, P = 0.069, four crosses sired by (02WF01x01WM05)1, P = 0.0002]. The results from all hybrid and control crosses of both types are summarized in Table 4. There is clear evidence that sex determination is disrupted in hybrid crosses. More specifically, mothers that produced only female progeny when crosses to their own species produced progeny of both sexes when crossed to foreign males. It is worth noting that when the hermaphrodites are excluded, the numbers of females and males from hybrid crosses are not statistically different from equal (112 versus 103, P = 0.539). Another observation is that hermaphrodites are ten times more frequent in hybrid crosses than in controls (chi-square 26.511, DF = 1, P = 0.000).

**Table 4:** Summary Showing Sex Ratio and Mitotype Content of Progeny Pooled by Cross Type.

Sex Ratio Bias of Mother	Type of Cross	Progeny Type	Number of Crosses Pooled	Number of Female Progeny	Number of Male Progeny	Number of Hermaphrodite Progeny	Number of Progeny with F mitotype	Number of Progeny with F+M mitotype
Female	E x E	PD	7	138	0	3	141	0
Female	E x T/H	HD	10	112	103	57	272	0
Female	E x E	PT	5	0	107	0	107	0
Female	E x H	HT	4	0	57	1	58	0
Male	E x E	PD	5	9	79	6	16	78
Male	E x T/H	HD	5	0	44	1	7	38
Male	E x E	PT	4	0	30	2	6	26
Male	E x H	HT	2	0	25	0	9	16
Total Female Bias			26	250	267	61	578	0
Total Male Bias			16	9	178	9	38	158

P: pure-species, H: hybrid; D: diploid, T: triploid.

**Figure 1:**
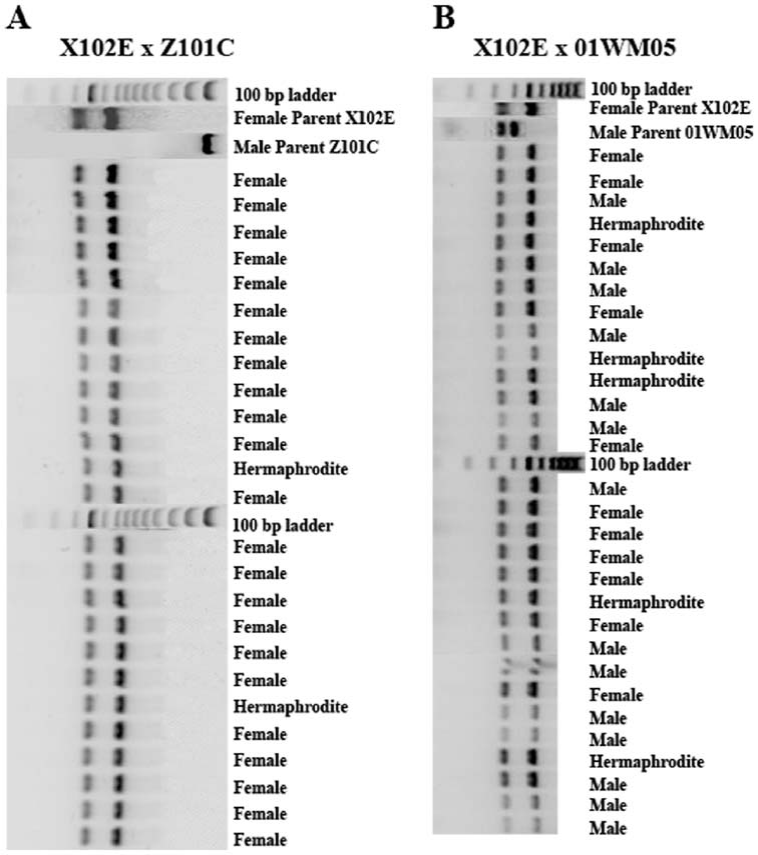
mtDNA content and sex of progeny of a female-biased mother crossed with a male of its own or of a different species. A) Homospecific cross. All progeny, but two, were females and all carried only the mother's mtDNA. B) Hybrid cross. Sex distribution among progeny is different than in A (11 progeny females, 14 males and 5 hermaphrodites), yet all progeny carry only the mother's mtDNA, as in A.

An entirely different result was obtained when the mtDNA content of progeny was examined. No progeny from the control crosses or the hybrid crosses contained the paternal genome, irrespectively of sex (Table 2, summarized in Table 4). Figure 1 provides an example. The figure shows the profile of the amplified COIII fragments after restriction with *EcoR*I. The first part (A) contains the profiles of all 25 progeny of the control cross X102E x Z101C, together with the profile obtained from the gametes of the two parents. It can be seen that all progeny (23 females and two hermaphrodites) contained only the mtDNA of the egg, as expected from a species with DUI. Part B shows the profiles of the 30 progeny of the hybrid cross X102E x 01WM05, a cross that shares the same female parent as the control cross of part A. Again, the 11 female progeny contained only the mtDNA of the egg, but this was also the case for the 14 males and the 5 hermaphrodites, quite contrary for a species with DUI. That sons produced by female-biased mothers contain no paternal mtDNA is a remarkable observation. It suggests that in mussels whether an offspring will inherit the paternal mitochondrial genome does not depend on its sex, but rather on its female parent. Female-biased mothers will produce offspring with no paternal mtDNA genome, irrespectively of whether these offspring will be only females, as is the case in pure-species crosses, or a mixture of females, males and hermaphrodites, as is the case in hybrid crosses.

### Sex and Mitotype of Progeny from Hybrid Crosses with Male-biased Mothers

There were five hybrid crosses with male-biased mothers and five control crosses (crosses HD and PD, respectively, Table 3). Three mothers were involved in both control and hybrid crosses. Unlike the case with female-biased mothers, the sex distribution was not different between hybrid and control crosses [mother 98A two control crosses, one hybrid cross, P = 0.072; mother (98AxWM8)A one control and one hybrid cross, P = 0.109; mother (98AxWM8)B one control and one hybrid cross, all progeny male]. The sex distribution was also not different in the three hybrid crosses sired by the same male. The results are summarized in Table 4. The sex distribution between control (type PD) and hybrid (type HD) crosses is nearly significant (chi-square 6.00, DF = 2, P = 0.050), yet the difference is minor, given that all crosses are dominated by males. Indeed, the difference is due to the fact that in hybrid crosses there are no female progeny, whereas female progeny appear in control crosses as a minority. The frequency of hermaphrodites in the two types of crosses is also non-significant (chi-square 1.127, DF = 1, P = 0.288).

**Figure 2:**
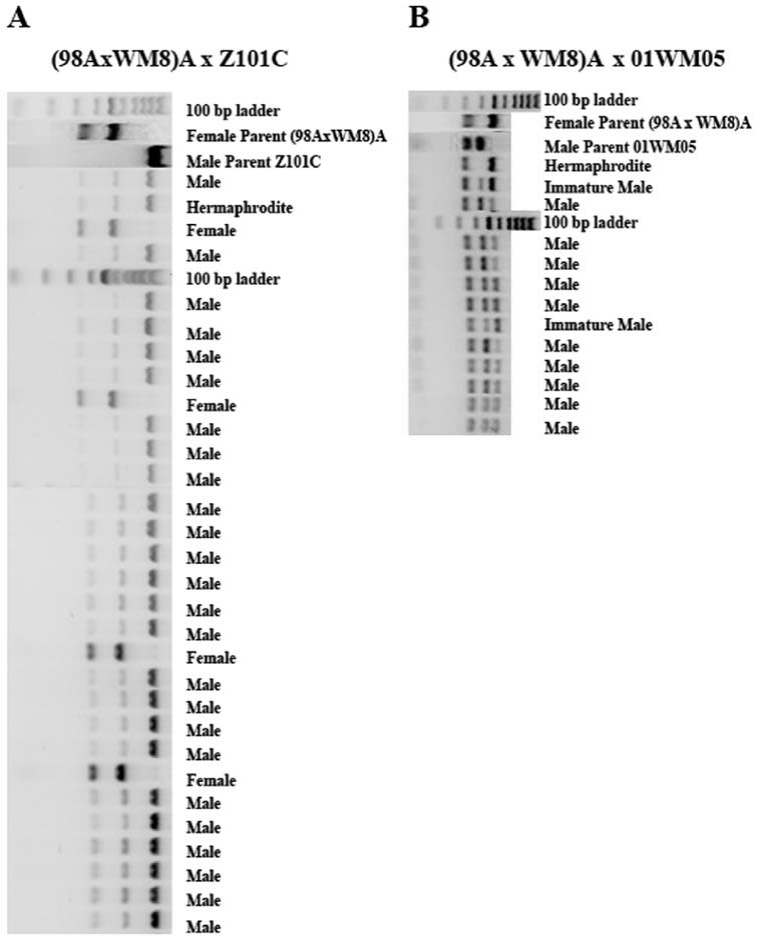
mtDNA content and sex of progeny of a male-biased mother crossed with a male of its own or of a different species. A) Homospecific cross. The majority of progeny are males (25 out of 30). These progeny and the single hermaphrodite carried the mtDNA of both parents. Four progeny were females and contained only the mother's mtDNA. B) Hybrid cross. All progeny, but one, were males and all carried the mtDNA of both parents. The single hermaphrodite carried only the mother's mtDNA.

There is no difference between control and hybrid crosses with regard to the mtDNA content of their offspring. Offspring with both parental mtDNA types are the majority in both types of crosses, averaging 83.5% (chi-square 0.047, DF = 1, P = 0.828). Figure 2 provides an example of the mtDNA content of progeny from a male-biased control cross (Part A) and a male-biased hybrid cross (Part B). The 25 male progeny and the single hermaphrodite of the control cross contained the mtDNA of eggs and sperm, but the four female progeny contained only the mtDNA of the egg, as expected from DUI. All progeny in the hybrid cross (part B) were males. These males contained both parental mitochondrial genomes, again as expected from DUI.

### Triploid Crosses

Progeny from crosses treated with cytochalasin B were tested for whether they were diploid or triploid. The gonads of all individuals that tested triploid were found to be of male-type, except three in which the gonad was of hermaphrodite type. This result agrees with the finding a previous study [17] that triploid mussels are males. This effect of triploidization applied to all our crosses regardless of whether the mother was female-biased or male-biased and regardless of whether the cross was pure or hybrid. The results are presented in Table 2 for female biased mothers (5 pure crosses, PT; 4 hybrid crosses, HT) and in Table 3 for female biased mothers (4 pure crosses, PT; 2 hybrid crosses, HT).

**Figure 3:**
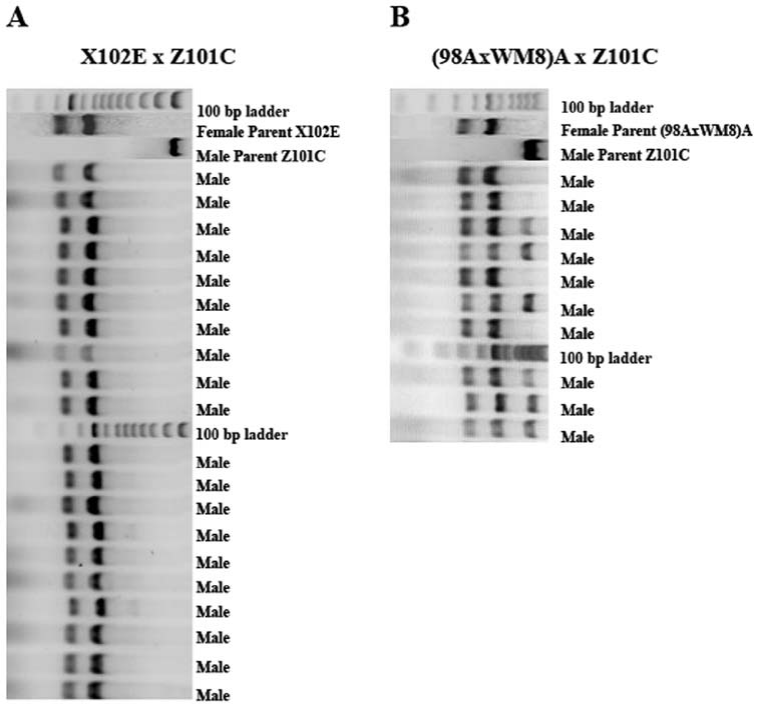
mtDNA content and sex of triploid progeny from two females crossed to the same male. A) Female-biased mother. All progeny were males, unlike with diploids from the same cross, where all but two were females (Fig. 1A). All progeny contain only the mother's mtDNA, as with diploids from the same cross (Fig. 1A). B. Male-biased mother. All progeny were males, as with diploids from the same mother (Fig. 2B). Of these, 6 carried the mtDNA of both parents and 3 carried only the mother's mtDNA.

Triploidization had no effect on what mtDNA genomes the offspring will inherit (Tables 2 and 3). As with diploid progeny, this depended entirely on the female parent. Triploid offspring of mothers that in control crosses produced only daughters contained only the F mitochondrial genome. Mothers that in control crosses produced a majority of sons (with both parental mitochondrial genomes) and a minority of daughters (with only the maternal mitochondrial genome) produced triploids that were all sons, among which a majority contained both parental mitochondrial genomes and a minority contained only the maternal mitochondrial genome. An example is shown in Figure 3, in which we give the restriction profiles of the triploid progeny from a cross in which the female parent produced female-biased progeny and of a cross in which the female parent produced male-biased progeny. The male parent was the same in both crosses. In both crosses all progeny were males. In the cross with a female-biased mother (Part A) all progeny contained only the maternal mitochondrial genome. In the cross with the male biased mother (Part B) some contained both parental mitochondrial genomes and some only the maternal one.

### Sperm mitochondria Segregation in Fertilized Eggs

**Figure 4:**
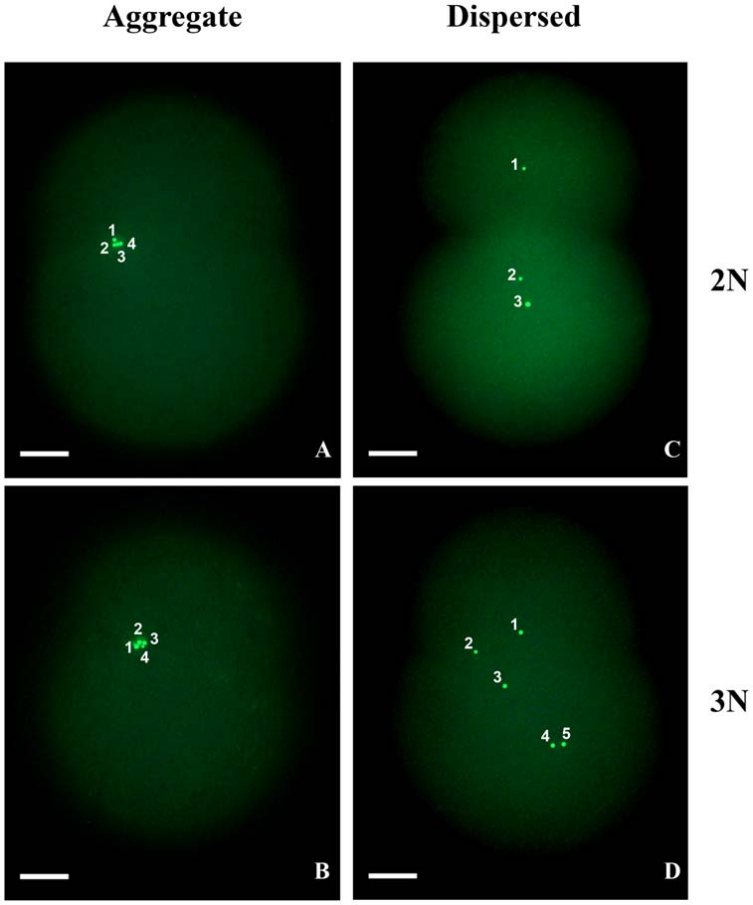
Sperm mitochondria segregation pattern in two-cell embryos. Numbering was overlaid on the original photos to indicate how many sperm mitochondria were visible. In the “aggregate” pattern sperm mitochondria form an aggregate and stay in the same blastomere. In the “dispersed” pattern sperm mitochondria disperse randomly in the two blastomeres. A and B: Eggs from 98B (a male biased mother), sperm from 00WM2. C and D: Eggs from X102E (a female biased mother), sperm from Z101C. 2N: diploid, 3N: triploid.

Cao et al. [10] and Cogswell et al. [11] used sperm stained with MitoTracker green, a florescent die that binds on the outer membrane of mitochondria, to follow the behavior of sperm mitochondria in mussel eggs. We have repeated this experiment using eggs from four female-biased and four male-biased mothers (Table 5). These eggs were fertilized by sperm from *M. edulis* (control crosses) or from a *M. edulis/M. trossulus* hybrid (hybrid crosses). Sperm stained with MitoTracker green was also used to fertilize eggs that were subsequently treated with cytochalasin B to induce triploidy. From each cross, we observed 16 to 40 two-cell embryos. All examined embryos, except two, from crosses with a female-biased mother showed the dispersed pattern, regardless of whether the cross was of a control (progeny type PD), hybrid (progeny type HD) or triploid (progeny type PT) type (Table 5). In crosses with a male-biased mother, the aggregate pattern was much more common, again regardless of the type of cross or the ploidy of the progeny (Table 5). An example for each ploidy class and sperm mitochondria segregation pattern is given in Figure 4. The conclusion from this experiment is that, like mtDNA inheritance, the pattern that the sperm mitochondria will form in the fertilized egg does not depend on the sex to which the embryo will develop or whether it would be diploid or triploid, but on the female that produced the egg.

**Table 5:** Mitochondrial Segregation Pattern in Pure and Hybrid Crosses of Diploid and Triploid *Mytilus*.

Cross Number	Parents	Type of Cross	Type of Progeny	Sex Ratio Bias of Mother	Number of Embryos with Aggregate Segregation Pattern	Number of Embryos with Dispersed Segregation Pattern
1	X102E x Z101C	ExE	PD	Female	0	16
2	X102E x (02WF01x01WM05)1	ExH	HD	Female	2	14
3	(X102Cx98WM4)A x (98AxZ101A)1	ExE	PD	Female	0	16
4	(X102Cx98WM4)A x (98AxZ101A)1	ExE	PT	Female	0	40
				Total Female Bias	2	86
5	(98AxWM8)a x Z101C	ExE	PD	Male	12	4
6	(98AxWM8)a x(02WF01x 01WM05)1	ExH	HD	Male	14	2
7	X102N x (98AxZ101C)2	ExE	PD	Male	32	3
8	X102N x (98AxZ101C)2	ExE	PT	Male	10	2
				Total Male Bias	68	11

E: *M. edulis*, P: pure-species, H: hybrid; D: diploid, T: triploid.

## Discussion

The distinguishing feature of DUI is that males transmit to their sons a mitochondrial genome different from the one females transmit to their progeny of either sex. As a result, there is a tight linkage between maleness and presence of the paternal mitochondrial genome. As we have stated in the Introduction, there was no firm evidence in the literature, up until this study, that these two features could be decoupled. The study we report here demonstrates that this is possible. We have found that females that produced exclusively daughters in homospecific crosses, produced daughters and sons in about equal numbers when crossed to heterospecific males. But the hybrid sons of these females contained no paternal mtDNA. The triploid sons of these same females also contained no paternal mtDNA. This is clear evidence that in mussels maleness and paternal mtDNA inheritance are separate phenomena. We may conclude that daughter-producing female mussels have two separate properties: 1) produce only female progeny and 2) prevent the transmission of the sperm's mtDNA to their progeny. Hybridization and triploidization interfere with the first property, but leave the second unaffected.

**Figure 5:**
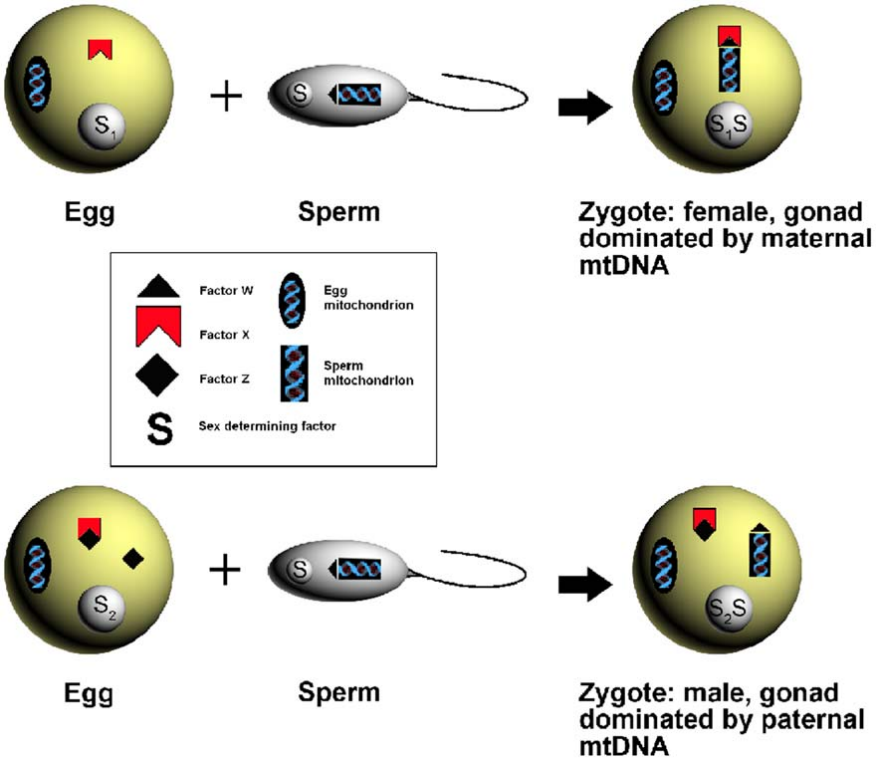
A model for sex determination and paternal mtDNA inheritance in mussels. Sex is determined by a nuclear locus S, with two alleles, S_1_ and S_2_, with different “dosage” effect (S_1_ = 1, S_2_ = 2). For maleness it is required that the dosage is 2 or higher. Only the maternal allele is expressed during the development of sex. The paternal allele is masked and contributes a dosage of zero, regardless of whether it is S_1_ or S_2_. Thus, sex is determined by the mother's genotype. The genotype of the male parent affects the sex of the progeny of his daughters. Paternal mtDNA transmission is affected by three nuclear genes. Locus W is male-expressed. It's product labels the outer surface of sperm mitochondria and differentiates them from egg mitochondria. Locus X is female-expressed. It supplies the egg with a factor that interacts with W and prevents sperm mitochondria from aggregating and co-segregating into the first germ cells. Locus Z is also female-expressed. It segregates for an active (Z) and an inactive (z) allele. It supplies the eggs with factor Z which suppresses factor X and allows sperm mitochondria to aggregate and co-segregate into the first germ cells. There is a tight linkage between Z and S_2_ and between z and S_1_ so that only the combinations ZS_2_ and zS_1_ may occur in an egg. Sperm mitochondria occur in the sperm's mid-piece, but are shown in the “head” for convenience. Egg mitochondria far out-number sperm mitochondria but we show one of each kind for convenience.

The decoupling of maleness and paternal mtDNA inheritance in mussels brings into new focus the mechanistic basis of DUI. Figure 5 is a modification of the original model we produced [27] as a working hypothesis about how DUI works. The novel element is the incorporation in the model of a sex-determining factor S, about which we make the following assumptions: a) it segregates for two alleles with different dosage effect, b) the paternal allele is inactive during the phase of sex determination, and c) for maleness, the dosage must exceed a certain threshold. Together, these postulates may explain why under normal circumstances the sex of a progeny is determined exclusively by the mother's genotype and why triploids develop as phenotypic males. It may also explain why hybridization and triploidization affect more strongly female-biased than male-biased crosses. In the latter, the eggs are already provided with the dosage needed for maleness, which is not affected by the inactive sperm allele.

With regard to the transmission of the paternal mitochondrial DNA the model remains as originally proposed. The W–X system can be considered analogous to the mammalian system that causes the destruction of sperm mitochondria and is apparently based on the ubiquitination of sperm mitochondria [28]. In mussels the equivalent of the destruction is the prevention of the sperm mitochondria to stay as an aggregate bound to the inner surface of the blastomere. These dispersed mitochondria will not find their way to the primordial germ cells of the embryo. Instead, they will follow a stochastic pattern of distribution in the cells of the developing organism, where they will remain a small minority compared to the population of egg mitochondria or may even be lost from the organism. This course of events will change if the egg is provided with the factor Z. In these eggs the X factor will be suppressed. Sperm mitochondria will form an aggregate and will be diverted to the embryo's germ cells. Within the germ line the paternal mtDNA will become dominant, so that when gametes are produced they will contain only the paternal mtDNA. According to Garrido and Gallardo [29], during spermatogenesis the immature spermatocytes undergo a loss of the majority of their cytoplasm and an actin-mediated reorganization of mitochondria, which apparently contain only paternal mtDNA.

To account for the coupling between sex and mtDNA inheritance the model postulates a tight linkage between the active allele of locus Z and the high dosage allele of locus S (haplotype ZS_2_) and between the null allele of locus Z and the low dosage allele of locus S (haplotype zS_1_). The linkage can be “physical”, i.e., loci Z and S are very closely located. Alternatively, it may emerge as a pleiotropic effect that can be mediated through various mechanisms, such as alternative splicing or genomic imprinting. In both cases it would be possible for the mechanism that controls mtDNA inheritance to remain unaffected when the mechanism for sex determination is disrupted by incompatibilities between genes from different species or by developmental anomalies caused by triploidization. There is, whoever, a “snag” in the model and it has to do with the timing of expression of the Z and S genes. Mussel females are of three types, those that produce almost exclusively daughters, those that produce sons in very high proportion and those that produce the two sexes in intermediate ratios. According to the model, the latter are heterozygous for the Z and the S loci. If we assume that the Z allele is expressed some time before the eggs are released, then for a heterozygous female to produce two types of progeny, one with the father's mtDNA and one without it, we have to assume that she produces two types of eggs: eggs in which the amount of substance Z is, stochastically, enough to inactivate substance X (these eggs will produce adults with the paternal mtDNA) and eggs in which the amount is not enough (these eggs will produce adults without the paternal mtDNA). However, the S alleles of this female will be activated after fertilization and will lead either to a male (allele S_2_) or female (allele S_1_) offspring. This leads to four types of progeny, males and females with and without the paternal mtDNA. But of these only two (females without paternal mtDNA and males with both types of mtDNA) are normally observed in nature and laboratory crosses. At present, we can propose no sound mechanism that would make the functions of factors Z and S coincide in time.

The model may lead to several insights regarding DUI. We report here that the female's property to produce eggs that upon fertilization will show the aggregate or the dispersed sperm mitochondria pattern is not affected by hybridization or triploidization. This reinforces the conclusion that the pattern of sperm mitochondria segregation is intimately related to the fate of the sperm mtDNA in the developing embryo. At the same time, the observation that triploid embryos from female-biased mothers will develop into males, even though their mitochondrial pattern is dispersed, eliminates the possibility that the mitochondria segregation pattern has anything to do with whether the embryo will develop into male or female. The mechanism through which the sperm's mtDNA becomes the dominant form in the germ line may or may not be related to the mechanism through which the sperm mitochondria invade the gonad in the early developmental stages. One possibility is that under normal conditions the sperm mitochondria are the only ones to enter the first germ cells of the male embryo. The other, more likely possibility is that the first germ cells contain sperm and egg mitochondria and that during development of the gonad the paternal mtDNA becomes dominant and, eventually, the only mtDNA occupant of the germ line. It is tempting to hypothesize that the control region of the paternal mtDNA may have a role in this process of domination. This hypothesis stems from the observation that mitochondrial genomes with the F-type nucleotide sequence but with a mosaic control region, i.e., a region that contains F-type and M-type control elements, follow the paternal line of transmission. However, it is not yet clear if all genomes with mosaic control regions are exclusively inherited through the sperm and, indeed, in *M. trossulus* populations from the Canadian Atlantic the maternal genome has a mosaic control region [30]. We simply do not have at present enough information to produce a more specific hypothesis for the second phase of the DUI mechanism, the one that is responsible for the exclusion of the maternal genome from the male germ line.

The purpose of this study was to gain an insight about the mechanism of DUI through experimental manipulation of pair-matings. The results have helped us produce a new version of a previous model about the mechanistic basis of DUI. The model is compatible with most observations we have about DUI, particularly in mussels, but also contains several assumptions about which we have at present no supporting evidence. At the same time, it helps redefine the aspects, experimental or theoretical, that should form the priorities for future research on this issue. The postulated silencing of the paternal sex-determining gene is an intriguing example for further experimental work. At the theoretical front, DUI has been seen, since its discovery, as a good example of intergenomic conflict [31], [32]. The work we present here highlights this conflict: it is to the “interest” of the maternal mtDNA genome that mussel females producing mostly daughters be more common in the population than females producing mostly sons. The paternal mtDNA genome has an opposite interest. Nuclear genes' interest is that neither of the two types becomes overly common. The tight linkage that we propose here between the alleles of a sex determining locus and the alleles at a locus that controls the transmission of paternal mtDNA might be the solution that the nuclear genome has imposed on the system. It is important to stress at this point that maleness was studied here as a “phenotype” inferred from gonad development. Triploids are most certainly sterile and males from hybrid crosses may also be largely sterile. Information from laboratory crosses on this issue is still lacking. In surveys of wild populations by Saavedra et al. [33] in Atlantic Canada, the area from which the animals used in this study originated, mtDNA introgression between these two species is rare and it may not go beyond post-F_1_ male hybrids. The possibility that the paternal mtDNA is, in some way, important for male fitness cannot be dismissed.

## Materials and Methods

### Species Identification and Mitotype Characterization

Mussel broodstock were selected from previously spawned animals held at the Bedford Institute of Oceanography, Dartmouth, Nova Scotia in order to choose females with known sex ratio bias [9]. Sperm or eggs were collected from the broodstock at spawning, except for one female (female 98A) from which a sample of the mantle tissue was taken, and used for the extraction of DNA for the identification of the species status (*M. edulis, M. trossulus* or hybrid) and the determination of the mtDNA content of the gametes of each brooder. DNA was extracted using a DNeasy kit (Qiagen) according to the manufacturer's instructions. DNA was quantified with PicoGreen (Invitrogen) using a FLUOStar Optima (BMG LabTech) and normalized to 10 ng/µL with 10 mM Tris (pH 8.0) for use in the PCR reactions.

Four nuclear DNA markers diagnostic for *M. edulis* and *M. trossulus* were used for species identification: ITS [34], Glu-5′ [23], MAL-I [35], and PLIIa [34], but only the first two could be consistently scored. The ITS primers amplify the ITS-1, 5.8S, and ITS-2 regions of rDNA and the Glu-5′ primers target a polyphenolic adhesive protein. The Glu 5′ product varies in size between the two species. The ITS product was digested with restriction enzymes to yield species-specific DNA fragments. Details of the protocols used to assess these markers are provided in Protocol S1.

Mitochondrial haplotypes were determined from the restriction fragment profile of an amplified segment of the cytochrome oxidase gene (COIII) after restriction with *Eco*RI and *Acc*I [33]. The fragment from the F genome of *M edulis* and the M genome of *M. trossulus* has an *Eco*RI restriction site, which is absent from the M genome of *M. edulis*. The inverse is true for *Acc*I. Control regions of mitochondrial genomes were determined by using the primers developed by Mizi et al. [36]. The ssFdl primer pair amplifies from the F mitochondrial genome of *M. edulis*, but not from the M mitochondrial genome of either *M. edulis* or *M. trossulus*. The ssMdl pair amplifies from the M mitochondrial genome of *M. edulis*, but not from the F mitochondrial genome of *M. edulis* or the M mitochondrial genome of *M. trossulus*. The identification of the control region was necessary to avoid using as parent a male with a masculinized (F-type) mitochondrial genome. The combination of the COIII and the control region assay make the determination of the mtDNA content of eggs or sperm unambiguous. Details of the protocols for assessing these markers are provided in Protocol S2.

### Production of Crosses and Rearing of Progeny

All crosses (Tables 2 and 3) were performed and reared at the Bedford Institute of Oceanography, Dartmouth, Nova Scotia, Canada. Details of the methods used to spawn, induce triploidy and rear progeny are provided in Protocol S3.

### Induction and Confirmation of Ploidy

Triploidy was induced by chemical means through the retention of the 2nd polar body of the developing embryo, producing an individual with a 2 maternal and 1 paternal chromosome complement. Flow cytometry protocols refined by Jackson et al. [37] and Cogswell et al. [38] were used to determine the success of triploidy induction in crosses whose eggs were treated with cytochalasin B. An initial screening was done on the larvae of each cross [38] to validate the success of the induction. Crosses with a high percentage of triploids were grown on to maturity. Somatic tissue samples of each of the mature progeny from these crosses were taken for flow cytometry at the same time as the gonad tissue was being prepared for histological determination of sex. ModFit LT Software (Verity Software House, Inc.) was used to normalize and differentiate 2N (diploid) and 3N (triploid) peaks.

### Visualization of Labelled Mitochondria in Mussel Embryos

The first division of fertilized eggs was completed in approximately 60–75 minutes post-fertilization. Embryos at this stage were placed on glass slides under cover slips. The fluorescing mitochondria were observed under a Nikon (Japan) E800 epifluorescence microscope equipped with a 450 to 490-nm bandpass excitation and a 520-nm longpass emission filter block set [10], [11]. The sperm mitochondria pattern was scored according to Cogswell et al. [11] as either dispersed (mitochondria found in both cells at the 2 cell stage), or aggregated (mitochondria in one cell, forming an aggregate on or near the cleavage furrow).

### Sex Determination of Progeny

The sex of the mussel progeny was histologically determined after approximately one year post-fertilization. For histological determination of sex, progeny were dissected using a sharp scalpel inserted between shell halves on the ventral side, slicing through the posterior and anterior adductor muscles and the hinge ligament. A thin 2–3 mm transverse section was cut, which included parts of the left and right gonad and also of the gill, foot and digestive gland [39]. Sections were placed in pre-labeled tissue microcassettes and placed in Davidson's Solution for 24–48 h [40]. For a visual sex determination and cross-reference with the histological examination, a small piece of gonad was cut at this stage from each mussel and examined under a light microscope for presence of sperm or eggs. Microcassettes were transferred to 70% EtOH and shipped to the Shellfish Health Unit of the Department of Fisheries and Oceans, Canada in Moncton, New Brunswick, where the samples were embedded in paraffin, put on to slides and stained with hematoxylin and eosin using standard techniques [39]. Fixed slides were sent back to the Bedford Institute of Oceanography (BIO) where they were scanned with a Nikon E800 Light Microscope under a 4x objective in a “search and rescue” pattern to determine presence of oocytes, or spermatocytes/spermatids. The progeny was characterized as female or male if only oocytes or only spermatocytes/spermatids could be found, and as hermaphrodite if both gametocyte types were found.

### Mitotyping of Progeny

A sample of the gonad was excised from each individual offspring during the dissection for histological determination of sex (see above) and frozen at −80°C. DNA was extracted and the mitotype of each individual was assessed using the COIII assay as described for the determination of the mitotype of parents.

## Supporting Information

Protocol S1 Details for the ITS and Glu5' Assays Used for Species Identification.
(0.03 MB DOC)

Protocol S2 Details of Assays Used for Identification of the mtDNA Types.
(0.04 MB DOC)

Protocol S3 Details Protocols of Spawning, Fluorescent Labelling of Sperm, Induction of Triploidy and Rearing of Progeny from Crosses.
(0.03 MB DOC)
