# Supplementary material for: Paternal mtDNA and Maleness Are Co-Inherited but Not Causally Linked in Mytilid Mussels
Source: PLoS One. 2009 Sep 11;4(9):e6976. doi: 10.1371/journal.pone.0006976 (PMC2736565; doi:10.1371/journal.pone.0006976)
Supplement: Protocol S1 — Details for the ITS and Glu5' Assays Used for Species Identification. (0.03 MB DOC) [file pone.0006976.s001.doc]

**Protocol S1. Details for the ITS and Glu5’ Assays Used for Species Identification.**

The PCR reaction for ITS consisted of 32.5 ng DNA, 1X PCR buffer (ammonium sulphate buffer (BioBasic Inc.)), 2mM MgSO_4_ (BioBasic Inc.), 0.2mM dNTPs, 0.2μM of each primer and 0.05U Tsg (BioBasic Inc.) in 15 μL . The PCR cycling conditions were as described in [34]. The PCR products were digested, without purification, in a 25µL reaction with a final concentration of 1X of the enzyme. The restriction solution contained 1U *Hha*I (TaKaRa Bio Inc.) with buffer M (TaKaRa Bio Inc.) incubated at 37°C for 6 h followed by a 20 min incubation at 65°C. Restriction fragments were size fractionated on 2% agarose using TAE buffer. The products were stained with SYBR Safe (Invitrogen) and visualized using a GeneGenius (Syngene) gel documentation system. The Glu5’ PCR reactions were setup in 10µL volumes containing 25ng of genomic DNA and 1X PCR buffer ((NH_4_)_2_SO_4_ buffer (MBI Fermentas)), 1.5mM MgCl_2_ (Fermentas), 0.2mM dNTPs, 0.2μM of each primer and 0.05U Taq (Fermentas); the PCR cycles were as described in [23]. The PCR products were visualized as described for ITS.
