# Supplementary material for: Paternal mtDNA and Maleness Are Co-Inherited but Not Causally Linked in Mytilid Mussels
Source: PLoS One. 2009 Sep 11;4(9):e6976. doi: 10.1371/journal.pone.0006976 (PMC2736565; doi:10.1371/journal.pone.0006976)
Supplement: Protocol S2 — Details of Assays Used for Identification of the mtDNA Types. (0.04 MB DOC) [file pone.0006976.s002.doc]

**Protocol S2. Details of Assays Used for Identification of the mtDNA Types.**

The COIII PCR reactions were setup to a final volume of 25μL (except when DNA amounts were limiting) containing 62.5ng genomic DNA, 1X PCR buffer (ammonium sulphate buffer (BioBasic Inc.)), 2mM MgSO4 (BioBasic Inc), 0.2mM dNTPs, 0.2μM of each primer (FOR1 and REV1 primers described in [33]) and 0.05U Tsg (BioBasic Inc.). When DNA amounts were limiting the COIII PCR was setup in a 10µL volume using 25ng of DNA with the concentrations of all the other components as described above. The PCR cycles consisted of an initial denaturation of 94°C for 2 min, 30 cycles of 94°C for 30s, 54°C for 30s and 72°C for 1 min, followed by 72°C for 10 min. Ten μL of the COIII PCR product was digested, without purification, with *Eco*R1 and *Acc*I (except where DNA was limited, in which case only the *Eco*R1 digest was performed) in a 15μL reaction as follows: 1X *Eco*R1 restriction digestion (MBI Fermentas) and 1.5U of *Eco*R1 for *Eco*R1 digests and Buffer 4 (NEB) and 1.5U of *Acc*I for *Acc*I digests. The restriction digests were incubated at 37°C for 6 h followed by 20 min heat inactivation at 65°C.

The ssFdl and ssMdl PCR reactions were setup in 10μL mixes containing 25ng genomic DNA, 1X PCR buffer (KCl buffer), 2mM MgCl_2_, 0.2mM dNTPs, 0.2μM of each primer and 0.05U Taq. For ssFdl, ssFdl1 and ssFdl2 see supplementary information in [36]. For ssMdl, primers were ssMdl1 5’-TAARTGAGGTTGGCTATAMGTGT-3’ (G. Rodakis, pers. comm.) and ssMdl2 [36; supplementary information]. For ssFdl the PCR cycle conditions were an initial denaturation of 94°C for 2 min, 35 cycles of 94°C for 30s, 58°C for 30s and 72°C for 1 min, followed by 72°C for 10 min. For ssMdl the PCR cycle conditions were an initial denaturation of 94°C for 2 min, 40 cycles of 94°C for 30s, 55°C for 30s and 72°C for 1 min, followed by 72°C for 10 min.

PCR products (ssFdl and ssMdl) or restriction fragments of PCR products (COIII) were stained and visualized as for Protocol S1. The COIII RFLP products were identified as Fed-1, Med-1, Ftr-1 or Mtr-1 (or combinations) based on the description of the restriction sites in Saavedra et al. [33]. ssFdl and ssMdl were categorized for the presence or absence of the ~180 bp product.
