# Supplementary material for: Paternal mtDNA and Maleness Are Co-Inherited but Not Causally Linked in Mytilid Mussels
Source: PLoS One. 2009 Sep 11;4(9):e6976. doi: 10.1371/journal.pone.0006976 (PMC2736565; doi:10.1371/journal.pone.0006976)
Supplement: Protocol S3 — Details Protocols of Spawning, Fluorescent Labelling of Sperm, Induction of Triploidy and Rearing of Progeny from Crosses. (0.03 MB DOC) [file pone.0006976.s003.doc]

**Protocol S3. Details Protocols of Spawning, Fluorescent Labelling of Sperm, Induction of Triploidy and Rearing of Progeny from Crosses.**

Selected broodstock were scrubbed clean using a fine bristled brush under cold domestic freshwater, wrapped in damp paper towel, then individually placed in labeled 250 ml polyethylene containers in a refrigerator at ~ 4^0^C overnight. The following day, mussels were removed from the refrigerator and each container was filled with 20^0^C 1µm filtered, UV-treated sea water. Animals were induced to spawn using thermal shock. The water in the containers was changed every 20-30 min alternating between 14^0^C and 20^0^C water until gametes were released. Upon retrieval of individual gametes, diploid crosses were initiated using quality eggs at densities of 20 eggs/ml and sperm at densities of 10-15 sperm/egg (to minimize the chances of polyspermy). Eggs and sperm were combined until all of the planned crosses were formed. Fertilization took place in 5 L buckets filled with 2 L of 15^0^C 1µm filtered, UV-treated sea water. Combined gametes were left undisturbed for ~30 min for fertilization to occur, then another 2 L of 15^0^C 1µm filtered, UV-treated sea water was added to each bucket to render its final volume.

Eggs intended for 3N induction were concentrated into individual 1 L beakers filled with 250 ml of UV treated, 1 µm filtered, 14°C (treated) seawater. In order to track the movement of the mitochondria within the developing embryo, some sperm were treated with the mitochondrial specific dye MitoTracker Green FM (MitoFM: Molecular Probes, Eugene, U.S.A.) following the protocols of Cogswell et al. [11]. MitoTrackerFM was diluted to a 1mM concentration in dimethyl sulfoxide (DMSO) (Sigma, Canada) shortly before spawning, added to sperm at a concentration 0.2µl/ml and incubated in the dark for 20 minutes at ~20°C. Eggs were then fertilized at a sperm density of 10-20 sperm/egg. At five minutes post-fertilization eggs were thoroughly rinsed with filtered sea water on a 20-µm filter to remove excess MitoTrackerFM and sperm that might be adhered to the egg membrane. Eggs not destined for fluorescent microscopy were fertilized with sperm at a concentration of 10-15 sperm/egg. For both cross types, starting 10 min post-activation and every 5 min thereafter, a small sample of fertilized eggs was examined microscopically to establish the point at which ~50% had released the first polar body. Based on concentrations suggested by Beaumont and Kelly [41] and Jackson et al. [37], at ~25 min post-fertilization eggs were treated with 125 µl of cytochalasin B (CB) (1 mg/ml CB in DMSO) in 250 ml of seawater to achieve a final concentration of 0.5 mg/l and 0.05% DMSO [38]. The fertilized eggs in the CB solutions were lightly plunged every 1-2 min to homogeneously expose eggs to CB over a 15 min treatment duration. Post-treatment, early embryos were gently poured onto a 20 µm Nitex screen and rinsed for 2-3 minutes with treated seawater. They were then exposed to 0.02% DMSO in seawater for 15 minutes to remove any persisting traces of CB [37]. After rinsing the eggs clean of CB, the developing zygotes from each cross were placed into a 250 L tank topped with treated seawater.

For both diploid and triploid crosses, after ~48 h of larval development from trochophore to D-stage, the mussel larvae were poured onto 40µm screens and examined using a microscope for health and survival. Densities were adjusted to 5 larvae/ml or 20,000 animals per bucket and fed a 50:50 algal mix of Tahitian *Isochrysis* and *Pavlova lutheri* at 35,000 cells/ml. Buckets were cleaned and water changed every second day. Once set, the mussels were fed a broader range of phytoplankton species at concentrations exceeding 35,000 cells/ml and placed in an upwelling unit to promote fast growth in order to reach sexual maturity the following year.
